# Psychological/behavioral interventions for emerging adults with chronic pain

**DOI:** 10.3389/fpain.2024.1253700

**Published:** 2024-02-27

**Authors:** Judith G. Foy, Sandra Kechichian, Michael R. Foy, Maisa Ziadni

**Affiliations:** ^1^ Department of Psychological Science, Loyola Marymount University, Los Angeles, CA, United States; ^2^ Graduate School of Education and Psychology, Pepperdine University, Malibu, CA, United States; ^3^ Department of Anesthesiology, Perioperative and Pain Medicine, Stanford University School of Medicine, Palo Alto, CA, United States

**Keywords:** emerging adult, chronic pain, psychological, pain intervention, mini-review

## Abstract

**Background:**

Emerging adults, of whom significant numbers report chronic pain, are characterized as having unique needs and challenges. Psychological/behavioral treatments found to be beneficial for reducing pain outcomes in children and adults are understudied in emerging adults. Following a systematic review of the literature, our objective is to report on quantitative studies of psychological/behavioral interventions for chronic pain in emerging adults.

**Method:**

We conducted a search of six databases (Cochrane Central Register of Controlled Trials, Google Scholar, ProQuest, PsycINFO, PubMed, and Web of Science) and reference sections in dissertations and systematic reviews to 4/29/2023. Keywords and phrases were search term combinations of “chronic/persistent pain”, “emerging/young adults,” and “intervention/treatment” using Boolean logic.

**Results:**

Our review resulted in identifying 37 articles, of which 2 duplicates were removed, and 31 were further excluded by a screening process based on various inclusionary and exclusionary criteria. The search yielded four studies on psychological/behavioral interventions (yoga, acceptance and commitment therapy and relaxation), all of which positively affected the pain experience and/or pain-related outcomes. These studies presented issues in design such as not being blinded or randomized, having a small sample size, and potential confounds that were not reported or examined.

**Discussion:**

The low number of studies reveals a large gap in the literature and is a call-to-action to further expand our understanding of effective and safer psychological/behavioral therapies for chronic pain in emerging adults. Successful pain management during this developmental phase may help young adults achieve positive trajectories for personal, occupational, relational, and health aspects of their lives.

## 1 Introduction

Development from the end of compulsory schooling (e.g., high school) to the onset of adult commitments (e.g., career) is known as emerging adulthood (18–29 years) (1). This stage is characterized by significant life transitions (1–3) in which personal, occupational, relational and health trajectories are determined (4, 5). Chronic pain is persistent pain lasting over 3 months (6). The impact of this type of pain on development and trajectories during emerging adulthood is not well understood. To our knowledge, there are no reviews of quantitative research on psychological/behavioral interventions in emerging adult samples with chronic pain.

Chronic pain affects a significant number of emerging adults, with prevalence rates ranging from 8.5% in the United States (7) to 11.6% worldwide (8), depending on sample and operational definitions of chronic pain (9). Several studies have found that prevalence rates for those attending university are higher compared to the general public. For example, 40% of university samples in Canada (10), 40.9% in Turkey (11), 54% in Norway (12) and 77.9% in Brazil (13) met criteria for chronic pain. In a recent systematic review of prevalence rates in university students, Serbic and colleagues concluded that the typical psychosocial stressors associated with college-life may exacerbate pre-existing chronic pain or even elevate the risk of chronic pain in individuals who were previously healthy (14). Depression and anxiety, higher in emerging adults compared to teens (12, 15), and in those who attend college vs. those who do not, are known associates of chronic pain (14). Stress and distress which are often experienced by typical college students, combined with perceptions of stigma and decreased help-seeking experienced by students with chronic pain may play a role in these enhanced prevalence rates (14).

Many college athletes experience chronic pain as a result of athletic injuries (16) and report higher levels of chronic pain and pain interference in their daily lives after college compared to their non-athlete alumni counterparts (17). Whereas pain perception in athletes is similar to that of non-athletes, in competitive situations, athletes may be less likely to report pain (18). However, outside of competition they are also more likely to seek help for pain than non-athletes (18) and may use different coping strategies compared to non-athletes (19). There is still much to be understood with regard to the elevated chronic pain rates in university students, whether they are athletes or not, and this cohort is in need of interventions for chronic pain.

Consequences of success or failure of pain management during emerging adulthood may be transformative. New freedoms associated with emerging adulthood may reduce positive physical and mental health outcomes in later adulthood (20, 21). Chronic pain can prevent emerging adults from achieving important goals and participating in experiences that might help them to become resilient over their pain, such as exercising and socializing (9, 22, 23). Alcohol, cannabis, and prescription opioid use and abuse escalates during this period (24–27). Mental illness/mood disorders are higher during emerging adulthood compared to adolescence, aged 12–17 years (28, 29), and co-occur more frequently with mental health problems during emerging adulthood than at other stages of development (30). Prescription opioids, self-medication, mental illness/mood disorders and substance abuse problems, relatively common and co-occurring, increase the risk of poor outcomes in adulthood for the emerging adult, whether they have chronic pain or not (29, 30). To better understand the complexity of chronic pain it is important to consider not only the biological factors involved, but psychological and social factors as well, currently integrated within a biopsychosocial model of health (31). Chronic pain affects relationships with peers, family, and medical professionals in ways that may have bidirectional effects on biopsychosocial and lifestyle factors (2, 9). Where persistent pain is linked to substance use/misuse, it can significantly impede psychosocial growth (32, 33).

Effective pain management during emerging adulthood helps to promote resilience and facilitate successfully taking on the roles and responsibilities of adulthood. Inadequate treatment of pain across age groups is a major risk factor for the development of persistent pain (34, 35). However, interventions that require a prescription and access to insurance are a barrier to healthcare in emerging adults, especially in the United States (5). Thus, many emerging adults with chronic pain report using non-pharmacological self-management/pain-coping strategies such as mindfulness meditation (36) and cognitive control (37), pharmacological substances such as cannabis (27, 38, 39), over-the counter (25) and non-prescribed medications (40, 41), as well as alcohol and tobacco (9). Nonmedical use of prescription opioids, reported by a third of a sample of high school seniors in the United States, is associated with worse outcomes in emerging adult years (41).

Pharmacological pain management during emerging adulthood may disrupt life trajectories in important ways that warrant further research. Opioid medications are the primary pharmacological treatment for this group, as they are for many others (25, 26, 42). However, opioid users report greater pain interference (negative effects on quality of life) than those not using opioids (25). Opioids also have serious side effects (43) that include an increase in the risk of hyperalgesia (heightened pain sensitivity) (44), and misuse/abuse disorders (38, 45).

While chronic pain poses significant challenges for emerging adults, it also presents unique opportunities for impacting life trajectories in positive ways. One of the characteristics of emerging adulthood is a sense of possibilities and optimism, where many potential futures await them (46). Nurturing habits can be learned during psychological/behavioral pain management, especially in college students, who presumably have easier and greater access to these types of services on campus (5).

There are several advantages of psychological/behavioral therapies relative to pharmacological treatments, including not necessarily requiring access to insurance/healthcare, a known difficulty for emerging adults (5). Although underreported in chronic pain patients, adverse events resulting from psychological/behavioral interventions are less serious than those from prescription medications (47). Interventions such as cognitive behavior therapy (CBT), acceptance and commitment therapy (ACT), mindfulness, and yoga have been shown to be effective for pain or pain-related outcomes in children and adults for some pain conditions (48–54). The most widely studied psychological/behavioral therapy, CBT, has been shown to potentially confer sustainable, healthy coping skills, even in self-administered (50) and single-session interventions (55, 56). Pain-related anxiety significantly and independently predicts opioid abuse problems in emerging adults (38), suggesting that evidence-based interventions targeting psychological factors of chronic pain may be especially effective. Interventions delivered via virtual reality (e.g., mindfulness) also show promise with younger children and older adults and appear to be quite effective for the management of anxiety (50).

## 2 Method

Because of the unique characteristics and needs of emerging adults, we sought studies that had explicitly examined a sample or performed sub-group analyses where the *M*_Age _± 1 *SD* was within 18–29 years. Keywords and phrases included in our search of the English language literature used combinations of “chronic/persistent pain”, “emerging/young adults,” and “intervention/treatment” using Boolean logic. Only interventions that were psychological/behavioral are reported here. Electronic searches using Cochrane Central Register of Controlled Trials, Google Scholar, ProQuest, PsycINFO, PubMed, and Web of Science databases were conducted up to 4/29/2023. In addition, reference lists for studies, dissertations, and systematic reviews were examined for relevant references.

Eligible studies had to meet the following criteria: (1) Include young/emerging adults between 18 and 29 years of age with chronic pain as the primary population or a subgroup that was analyzed separately. If emerging adults were only a proportion of the sample for a study, or if the range was not provided, the study was included only if *M*_Age _± 1 *SD* fell within the target range. The study needed to include participants who had recurrent, frequent episodic, persistent, or chronic pain, meeting International Association for the Study of Pain (IASP) requirements of pain lasting at least 3 months (6, 57). No attempt was made to include/exclude specific types of pain. (2) Clinical study of any design published in an academic journal (qualitative and quantitative studies, clinical trials). (3) Received treatment for chronic pain that included any psychological/behavioral non-pharmacological intervention, but not physical therapy, exercise, or pharmaceuticals (including cannabis/vitamins). (4) Include any pain measurement such as pain frequency, pain intensity, pain duration, pain interference and/or any pain-related outcomes with an empirically-validated relationship to pain such as analgesic use, sleep, pain-related quality of life, pain-condition symptoms and improvement [e.g., rheumatoid arthritis (RA) symptoms and RA symptoms improvement], mood, pain acceptance, fatigue, mindfulness, psychological distress, pain-related anxiety, or pain-related depression and (5) published in English.

Studies were excluded if they (1) described a majority of participants who were younger than 18 years, older than 29 years, or did not conduct separate analyses on emerging adults, (2) did not meet the criteria for chronic pain (≥3 months), (3) described a sample of emerging adults/university students learning about pain but not meeting criteria for chronic pain (e.g., as in a pain neuroscience education class), (4) none of the treatment groups received an intervention that was psychological/behavioral and (5) was primarily a prevalence or descriptive study without a treatment.

Two independent groups of undergraduate reviewers (two in each group) who were trained to 90% reliability (percent of agreement with expert reviewer for each review group) by one of the authors, screened articles at the title and abstract level. Articles where all of the criteria were met and/or those that the reviewers disagreed upon, were then screened by the senior authors (JF and MZ) and the reviewers discussed the full-text reports until consensus was reached.

## 3 Results

Search results up to 4/29/2023 using the above-mentioned search criteria included 37 articles. Of these, two duplicates and 31 studies not meeting our criteria for inclusion were removed by the two independent groups of reviewers who screened abstracts. Four articles were excluded after review of the full reports because they were outside our target age range, involved a survey but not treatments, and the interventions were not psychological/behavioral.

Four quantitative studies on psychological/behavioral treatments for chronic pain in emerging adults (58–61) were identified (see Table 1). Two were on effects of yoga, and another on ACT, both of which demonstrated efficacy in other age-groups (53, 54). Another study (61) was on the effects of autogenic relaxation training (AT), which was used as a comparison condition for physical therapy, an excluded treatment for this review. AT has limited but promising support in other age-groups (65). These studies were published from 2013 onwards, although the literature search was open-ended.

**Table 1:** Characteristics of psychological/behavioral pain management studies for emerging adults.

Study	Sample size	Pain source	Intervention, comparisons, follow-ups (where relevant)	Pain and/or pain-related outcomes	Statistically significant findings
Álvarez-Melcón, et al., (62)	*n *= 76 (relaxation group); *n *= 76 (relaxation + PT group); 18–25 years; 55.3% female, 44.7% male	Frequent episodic or chronic tension-type headache (TTH)	Relaxation (Autogenic Training: AT), randomly assigned (control group but treatment of interest in the present mini-review), Comparison = relaxation + physical therapy (experimental group); baseline, post-treatment (4 weeks after treatment), and one follow up (FU) 3 months after treatment	Self-report measures: Frequency of headache (days/4 weeks), intensity of headaches (VAS: 0–10), duration of headaches (h/day), analgesic use (days/4 weeks)	Pre-Post Treatment Comparisons Relaxation: Significant improvements in frequency (*F* = 26.41***, $\eta_{p}^{2}=.50$), duration (*F* = 5.19*, $\eta_{p}^{2}=.22$, and analgesic use at 3 months (*F* = 13.29***, $\eta_{p}^{2}=.28$). *F* range: 5.19–26.41 *p* range:.024–.001 $\eta_{p}^{2}$ range:.11–.50 Clinically significant (63, 64) improvements in relaxation group: Frequency*:* 32% moderately clinically significant difference Duration: 22% minimally clinically significant difference Analgesic use: 29% minimally clinically significant difference Between-groups at 3 months Lower frequency in Combined group than Relaxation group *t *= 2.61**, *d *= .42, Lower intensity in Combined group than Relaxation group *t *= 2.77**, *d *= .44. *t* range: 2.61–2.77 *p* range:.01–.006, *d* range:.42–.44
Evans et al., (60)	*n *= 26; 16–35 years, 100% female, 0% male	Rheumatoid arthritis (RA)	Yoga vs. Treatment as Usual waitlist control group (TAU) randomly assigned; Baseline vs. post treatment vs. 2 months follow-up. Length of treatment of 6 weeks (12 sessions)	Self-report measures: Health-related quality of life (vitality, bodily pain, general health, and mental health subscales of SF-36), pain disability index functioning (PDI), arthritis-specific symptoms and functioning (HAQ- DI, DAS, GIS), mood (somatization, depression, anxiety, global severity subscales of BSI), fatigue (FACIT-Fatigue), pain acceptance (CPAQ), mindfulness (FFMQ), arthritis self-efficacy (ASES), and weekly ratings of worst pain, average pain, anxiety, and depression (WMF)	Pre-Post Treatment Comparisons at 2-month follow-up: Yoga group had improvements in quality of life general health (*F* = 4.06**), pain disability (PDI) (*F* = 3.93*, *p* = .03 ); WMF decrease for worst pain (*F* = 2.63, *p* = .01), WMF depression (*F* = 3.29, *p* = .001) and WMF anxiety (*F* = 4.39, *p* = .001). *F* range: 2.63–4.39 *p* range: .03-.001 Clinically significant improvements in treatment group (IMMPACT^b^): SF-36 pain, general health and vitality subscales, BSI somatization and global severity, fatigue, and arthritis self-efficacy Between-groups: Improvements in yoga participants compared to TAU in SF-36 general health (*F* = 8.16**) and vitality (*F* = 9.79**) subscales, HAQ arthritis-general health subscale (*F* = 4.45*), fatigue (*F* = 7.99*), BSI (mood) somatization (*F* = 7.99*) and global severity (*F* = 4.36*) subscales, chronic pain acceptance (*F* = 8.77**), non-judging facet of mindfulness (*F* = 4.37*), pain self-efficacy (*F* = 4.86*), RA symptoms (GIS) (*F* = 3.29**) *F* range: 3.29–9.79 *p* range: .05–.01
Evans, et al., (61)	*N *= 21 in Rome III adult criteria [young adult (YA) group]: (11 in yoga group and 10 in TAU group) 18–26 years, 70% female, 30% male	Irritable bowel syndrome (IBS); recurrent abdominal pain or IBS using Rome III adult criteria	Yoga vs. TAU; randomly assigned; Baseline vs. post-treatment vs. 2 months follow-up (FU) Treatment length of 6 weeks (12 sessions)	Self-report measures: Symptom improvement (GIS), abdominal pain intensity (NRS), IBS symptoms (abdominal symptoms subscale of CSI), health-related quality of life (physical functioning subscale of SF-36), physical and psychosocial perceived difficulty in physical and psychosocial functioning in previous 2 weeks because of physical health (FDI), psychological distress (BSI-18), physical and functional consequences of fatigue (Facit-Fatigue), sleep quality in last month (PSQI), and weekly ratings of worst pain, constipation, nausea, diarrhea (WMF)	Pre-Post: *WMF*: Improvements over time for global improvement, (*F *= 3.37**), worst pain (*F *= 2.16*), constipation (*F* = 2.52*), nausea (*F *= 2.12*). *F* range: 2.12–3.37, *p* range:.04–.001 Clinically significant improvements in yoga group(s): 27% minimally clinically significant difference in global improvement in symptoms, compared to control, $\chi^{2}=11.13$, *p* = .03 (63) Between-groups Improvements in YA yoga group compared to control group in IBS symptoms, *F *= 5.68*, $\eta_{p}^{2}=.25$, EMM = 10.97, global severity, *F *= 5.03*, $\eta_{p}^{2}=.228$, EMM = 13.06, functional disability = 5.06*, $\eta_{p}^{2}=.23$, EMM = 11.40, sleep quality, *F *= 6.16*, $\eta_{p}^{2}=.266$, EMM = 1.35, and fatigue, *F *= 4.29*, $\eta_{p}^{2}=.2.02$, EMM = 30.57 *F* range 4.29*–6.16* p range:.05–.02 $\eta_{p}^{2}$ range: 2.02–2.66
Martin et al., (65)	*n *= 10, 12–20 years, 60% female, 40% male	Chronic Pain associated with Neurofibromatosis type 1 (NF1)	Acceptance and Commitment Therapy (ACT), baseline vs. follow-up at 3 months; initial workshop = 2 days, booster session 4–6 weeks later. FU at 3 months, outcomes were measured through mail.	Self-report measures: pain interference (MPBI and PII), pain intensity (McGill VAS), functional disability (FDI), pain acceptance (CPAQ-A), pain-related anxiety (PASS-20), pain-related depression (CES-D), health-related quality of life (IPI), psychological distress (BSI), techniques patients use to manage pain (PMI), NF disease severity (NFDS)	Pre-Post (patient results only reported here): Decline in pain MPBI interference (*p* = .04) and McGill pain intensity VAS (*p* = .01) at 3 months compared to baseline *p* range:.04–.01 (Wilcoxon values not available) Clinically significant improvements in treatment group: N/A Between-groups: N/A

JBI Checklists Joanna Briggs Institute (66).

ACT, acceptance and commitment therapy; ASES, arthritis self-efficacy scale; AT, autogenic training; BSI-18, brief symptom inventory; CES-D, center for epidemiological studies depression scale; CPAQ, chronic pain acceptance questionnaire; CPAQ-A, chronic pain acceptance questionnaire—adolescent report; CSI, child somatization inventory; DAS, disease activity scale; ES, effect size; EMM, estimated marginal mean; F, F-ratio; FABQ, fear-avoidance beliefs questionnaire; FACIT-Fatigue, functional assessment of chronic illness therapy fatigue subscale; FDI, functional disability; FFMQ, five factor mindfulness questionnaire; FU, follow-up; GIS, global improvement scale; HAQ- DI, health assessment questionnaire disability index; IBS, irritable bowel syndrome; IPI, impact of pediatric illness scale; N/A, not available; NF1, neurofibromatosis type 1; NFDS, NF disease severity; NRS, numeric rating scale; MPBI, modified brief pain inventory; ODI, K-Oswestry disability index; PASS, pain anxiety symptoms scale; PDI, pain disability index; PII, pain interference index; PMI, pain management inventory; PSQI, pittsburgh sleep quality index; PROMIS, patient-reported outcomes measurement information system; PT, physical therapy; RA, rheumatoid arthritis; RMD, K-Roland–morris disability; SF-36, short-form 36 item health survey); t, *t*-test; TAU, treatment as usual; TTH, tension type headache; VAS, virtual analogue scale; WMF, weekly monitoring form; YA, young adult.

* *p *< .05.

** *p* < .01.

*** *p *< .001.

Each of the studies met our inclusion criteria requiring that the *M*_Age _± 1 *SD* were within 18–29 years, although none of them used the term “emerging adult.” Instead, the term “young adult”, and in the exclusively female study, “young women” was used. Intervention fidelity was monitored to various degrees in all of the studies: weekly monitoring forms (58, 59), weekly follow-up sessions (61), or about 1.5 months before the final assessment at 3 months (60).

All of the studies had small samples focused on one pain type: rheumatoid arthritis (58), irritable bowel syndrome (59), neurofibromatosis type 1 (60), and tension-type headache (61). A variety of chronic pain definitions was used; three of them were clinical definitions for the specific diagnosis (58, 59, 61), while the other study relied on self-report of chronic pain to a nurse practitioner during a physical examination at their most recent visit to the clinic (60).

Random assignment was used in three studies with waitlist treatment-as-usual control groups (58, 59) or an active comparison condition (61). In the ACT study, comparisons were made to the baseline values of the outcome measures. All studies included follow-ups which occurred at two months (58, 59), three months (60), and two assessments at four weeks and three months (61).

At least some concurrent pharmacological treatments were reported in all the studies, but only the relaxation study (54) also reported other nonpharmacological treatments. Two of the studies (58, 59) reported comorbid conditions in their participants.

The studies were conducted in yoga studios (58, 59), in-person clinics (two-day workshop), participants' homes (61), and booster sessions by telephone (60). Interventions were provided by experienced/senior teacher advisors in the yoga studies (58, 59), or specialized therapists (60, 61).

The IASP definition of pain requires that it be assessed in multiple domains, including reports of an individual's experience of pain (62, 67) and include behaviors expressed or not expressed in various clinical and research contexts (67, 68). All the studies assessed more than just reported pain intensity (e.g., visual analogue scale). As shown in Table 1, self-reported pain and pain-related outcomes in these studies included frequency, intensity, and duration of symptoms, as well as ratings of worst pain and average pain, pain functioning, and pain interference. Also included were measures of analgesic use, health-related quality of life, pain acceptance, pain efficacy, mood, anxiety, fatigue, sleep, and mindfulness. Notably, the measures used in the yoga studies (58, 59) assessed the experience of pain (worst pain and average pain in weekly ratings) as well as measures of depression and anxiety, well known to be linked with pain measures (69).

Each of the interventions showed statistically significant effects on pain experiences and several also showed statistically significant results for the secondary measures as shown in Table 1. Pre-post and between-groups findings were reported in the randomized-control trials, which also reported on the clinical significance of findings. The yoga studies (58, 59) used the Initiative on Methods, Measurement, and Pain Assessment in Clinical Trials (IMMPACT: http://www.immpact.org) recommendations (63). One study (61) reported clinical significance of the visual analogue scale (VAS) according to established criteria.

All the studies used designs graded as level II or moderate quality of evidence, a reasonable indication that future studies will replicate this finding (65). Methodologic limitations included internal and external validity issues according to the framework established by the United States Preventive Services Task Force (70), as described in Table 2. Some of these limitations, but not all of them, are typical challenges in psychological/behavioral interventions where participants actively participate in therapy. Internal validity was negatively affected by lack of participant and provider blinding to treatment condition, omission of details on concurrent pharmacological and non-pharmacological pain management, and the potential confounding effects of which were not described and/or analyzed statistically among other flaws. Only two of the studies (59, 61) reported *a priori*-sample size calculation. Limitations to external validity (Table 2) included small samples. Stringent exclusionary criteria regarding pain type and pain source restricted diversity in the samples. A lack of diversity in providers and settings in which the therapies were conducted also somewhat limit the generalizability of these studies.

**Table 2:** Design and methodologic rigor for studies of psychological/behavioral interventions in emerging adults.

Study	Level of evidence GRADE	Design	Internal validity JBI and USPTF	External validity JBI and USPTF	Limitations
Álvarez-Melcón et al. (62)	II	Randomized Control Trial (Parallel Groups)	Fair	Fair	Internal validity: Inability to blind participants and providers to intervention; effects of other treatments including medication not provided; possible confound (baseline male-female ratio difference between groups) not controlled for (note-combined had higher female so unlikely to influence results reported); intervention fidelity not provided. External validity: Only generalizable to college students with TTH diagnosed by a doctor and meeting IHS criteria, and without high state-trait anxiety. Results may not generalize to other types of relaxation training or to other therapists.
Evans et al., (60)	II	Randomized Controlled Trial (Parallel Groups)	Fair	Fair	Internal validity: Inability to blind participants and providers to intervention; effects of other treatments including medication and yoga practice at home not provided; effects of other medical conditions not provided; possible confound (baseline difference in RA duration) was statistically controlled; attendance during intervention not provided; 10 practiced at home during intervention, at FU 6 still practicing yoga. External validity: Small sample size, results only generalizable to females with RA for at least 6 months, to Iyengar yoga, with reported sequences of yoga poses, and to yoga in a college yoga clinic.
Evans, et al., (61)	II	Randomized Controlled Trial (Parallel Groups)	Poor	Fair	Internal validity: Inability to blind participants to their treatment assignment; lack of an active control group; differential attrition between groups; effects of other treatments including medication and yoga practice in TAU group not provided; effects of other medical conditions not provided; number of sessions attended by YA was 8.8/12 (73.33%); low rate of home practice at 2-month follow-up. External validity: Small sample size, results only generalizable to patients with IBS, to Iyengar yoga with reported sequences of yoga poses, and to yoga in a college yoga clinic.
Martin et al., (65)	II	Quasi-experimental	Poor	Poor	Internal validity: Inability to blind participants to their treatment assignment; lack of control group; only one measurement per period, treatment fidelity reported at 60% at least weekly use. External validity: Small sample size; only 64% of families agreed to participate; results only generalizable to patients with NF, to manualized ACT treatment in college classrooms or public settings

ACT, acceptance and commitment therapy; GRADE, grading of recommendations assessment, development, and evaluation; IBS, irritable bowel syndrome; IHS, international headache society; IMMPACT, methods, measurement and pain assessment in clinical trials; JBI, Joanna briggs institute; NF, Neurofibromatosis; USPTF, U.S. preventive services task force; RA, rheumatoid arthritis; TAU, treatment as usual; TTH, tension type headache.

## 4 Discussion

The lack of intervention research in emerging adults with chronic pain is due, in part, to the fact that this population is challenging to recruit and access (5). The reasons for this are complex, and include young adults' reluctance to self-identify as having chronic pain, especially if they are college athletes (18). Additionally, the perception of stigma appears to drive a lack of help-seeking behaviors within this cohort (71). Effective recruitment of large samples of emerging adults, attending as well as not attending institutions of higher education, is needed.

Although all of the studies reported factors related to pain, future studies within this cohort should attempt to utilize a biopsychosocial model of health in their design: for example, biological (e.g., chronic pain in childhood, family history, sex), lifestyle (e.g., sleep, recreational drug use, physical activity), psychological (e.g., anxiety and depression, stress, pain catastrophizing, pain coping), social (e.g., history of abuse, parental mental health and family functioning) factors as well as those related to pain interference (e.g., restriction to daily activities, social, work and college participation (9). Perceptions of social stigma (72, 73), illness invalidation (71), and perceived injustice (74, 75), also related to the pain experience, should be addressed as well. Given that 5% of emerging adults in the United States identify as transgender/nonbinary, and that transgender individuals report higher pain sensitivity to induced mechanical pain stimuli than cisgender individuals (76), we recommend that future studies of pain treatments in emerging adults include information on both sex and gender identification.

The high prevalence rate of chronic pain in college students compared to the general public warrants further research. Understanding risk factors such as participation in college athletics (16, 77) and mental health vulnerabilities like depression and anxiety may help to develop interventions that can help mitigate or prevent persistent pain in emerging adults (14). Psychological interventions with known benefits to mental health in other age groups may help emerging adults with chronic pain whether or not they attend college. Also unknown is the prevalence of foregoing college or professional sports due to chronic pain and the possibility of differential rates of chronic pain in emerging adults who attend compared to those who do not attend college.

In recent years, the introduction of non-traditional mental healthcare services and tools (e.g., mobile apps, peer counselors, online support groups) has increased access to and utilization of behavioral health services (76, 78). For example, tele-health success is linked with the development of self-efficacy (79), a core need of emerging adults (1, 46). Virtual-reality (36, 50, 80), self-administration (50), and single-session practices (55, 56) may also be beneficial to emerging adults with chronic pain, though empirical studies to evaluate these non-traditional services are needed.

Several major limitations of the reviewed studies include small sample size, inability to blind participants, effects of other treatments including medication not identified, possible confounds not statistically controlled, lack of active comparison groups, and limited generalizability to other pain types (Table 2). Future recommendations from this analysis include the assessment of baseline differences between groups in pain studies (81), and the influence of moderating potentially confounding pharmacological substances and psychological/behavioral activities and treatments. An understanding of these critical factors is important to measure and study the effectiveness of different practices in helping control chronic pain in emerging adults.

## 5 Conclusion

Despite evidence for the effectiveness of psychological/behavioral interventions in other age-groups, especially for CBT (82), we found only four quantitative studies of such treatments for chronic pain targeting emerging adults (58–61), and none for CBT. Whereas these studies showed positive effects of the interventions (yoga, ACT, and relaxation) on pain outcomes, studies are needed that focus on a wider variety of therapies, diverse populations, and pain types. These studies/therapies must address the unique developmental needs (23, 83, 84) and barriers to accessing treatment (5, 9) in emerging adults. Non-traditional delivery models such as virtual-reality (36, 50, 80), self- administration (50), tele-health (79), and single-session modalities (55, 56) may be especially beneficial.

The stakes are high at both individual and societal levels as many important trajectories in life are established during emerging adulthood. Unfortunately, the road to second chances is narrow with respect to earning college degrees and obtaining jobs where individuals can be self-sustaining members of society. The most important influences on mental health in those with chronic pain are the person's ability to work, and psychosocial factors, such as pain catastrophizing, independent of type of pain and pain intensity (85). Psychosocial factors are highly amenable to improvement with psychological/behavioral therapies. A better understanding of resilience trajectories established by emerging adults with chronic pain and moderator influences, such as psychosocial factors on pain outcomes and pain-related experiences can lead to better access to effective therapies, greater utilization of them, and more effective pain management for emerging adults.
